# Metabolomic signatures of drug response phenotypes for ketamine and esketamine in subjects with refractory major depressive disorder: new mechanistic insights for rapid acting antidepressants

**DOI:** 10.1038/tp.2016.145

**Published:** 2016-09-20

**Authors:** D M Rotroff, D G Corum, A Motsinger-Reif, O Fiehn, N Bottrel, W C Drevets, J Singh, G Salvadore, R Kaddurah-Daouk

**Affiliations:** 1 Department of Statistics, North Carolina State University, Raleigh, NC, USA; 2 Bioinformatics Research Center, North Carolina State University, Raleigh, NC, USA; 3 Department of Drug Discovery and Biomedical Sciences, Medical University of South Carolina, Charleston, SC, USA; 4 UC Davis Genome Center, University of California Davis, Davis, CA, USA; 5 Department of Biochemistry, King Abdulaziz University, Jeddah, Saudi Arabia; 6 Department of Neuroscience, Janssen Research and Development, Titusville, NJ, USA; 7 Department of Neuroscience, Janssen Research and Development, San Diego CA, USA; 8 Department of Psychiatry, Duke University Medical Center, Durham NC, USA; 9 Duke Institute for Brain Sciences, Duke University, Durham, NC, USA

## Abstract

Ketamine, at sub-anesthetic doses, is reported to rapidly decrease depression symptoms in patients with treatment-resistant major depressive disorder (MDD). Many patients do not respond to currently available antidepressants, (for example, serotonin reuptake inhibitors), making ketamine and its enantiomer, esketamine, potentially attractive options for treatment-resistant MDD. Although mechanisms by which ketamine/esketamine may produce antidepressant effects have been hypothesized on the basis of preclinical data, the neurobiological correlates of the rapid therapeutic response observed in patients receiving treatment have not been established. Here we use a pharmacometabolomics approach to map global metabolic effects of these compounds in treatment-refractory MDD patients upon 2 h from infusion with ketamine (*n*=33) or its *S*-enantiomer, esketamine (*n*=20). The effects of esketamine on metabolism were retested in the same subjects following a second exposure administered 4 days later. Two complementary metabolomics platforms were used to provide broad biochemical coverage. In addition, we investigated whether changes in particular metabolites correlated with treatment outcome. Both drugs altered metabolites related to tryptophan metabolism (for example, indole-3-acetate and methionine) and/or the urea cycle (for example, citrulline, arginine and ornithine) at 2 h post infusion (*q*<0.25). In addition, we observed changes in glutamate and circulating phospholipids that were significantly associated with decreases in depression severity. These data provide new insights into the mechanism underlying the rapid antidepressant effects of ketamine and esketamine, and constitute some of the first detailed metabolomics mapping for these promising therapies.

## Introduction

Major depressive disorder (MDD) is a complex illness associated with profoundly elevated rates of socio-occupational disability, medical morbidity and mortality. In the US, ~17% of individuals develop MDD within their lifetime.^1^ Although many treatment options are available, most therapies require weeks to exhibit therapeutic benefit and many individuals who suffer from MDD remain treatment-resistant, emphasizing the need for more effective and more rapidly acting therapies.^2, 3, 4^ In addition, over half of patients treated with selective serotonin reuptake inhibitors or selective norepinephrine reuptake inhibitors fail to achieve full symptom remission.^5^ Ketamine has been proposed as a promising therapeutic intervention for treatment-resistant depression (TRD), due to its rapid-onset of antidepressant effects (within 4 h post administration), in subjects who previously did not respond to multiple treatment trials. Indeed, studies have shown up to a 71% response rate for ketamine administration in MDD and a up to a 64% response rate in patients with TRD.^6, 7^

Ketamine is an antagonist for the glutamate *N*-methyl-d-aspartate (NMDA) receptor,^8^ and is a racemic mixture of two enantiomers, *R*- and *S*-ketamine. The *S*-ketamine enantiomer, referred to as esketamine, is threefold more potent than the *R*-ketamine enantiomer and has been shown to be similarly effective for decreasing depression.^9^ Esketamine is currently being investigated in phase 3 clinical trials, underscoring the importance of the ketamine/esketamine mechanism of action in the treatment of MDD and TRD.

However, the mechanism by which ketamine results in a rapid decrease in depressive symptoms does not appear to be explained entirely by its antagonizing effect on NMDA receptor alone, as its antidepressant effects extend well beyond its half-life.^10^ In addition, the NMDA blockade has been putatively shown to trigger a complex downstream intracellular cascade, which included the release of brain-derived neurotrophic factor and mTOR activation, and eventually leads to dendritic remodeling and synaptogenesis.^10, 11, 12^

Pharmacometabolomics associates changes in endogenous metabolite levels to phenotypes, drug exposure and drug response.^13, 14^ Recently, pharmacometabolomics approaches have identified significant associations with other psychiatric disorders and therapies including insight into the mechanism of action and mechanism of variation of response to selective serotonin reuptake inhibitors, such as citalopram/escitalopram response in patients with MDD.^15^ In addition, pharmacometabolomics approaches have identified a putative role for the methoxyindole and kynurenine branches of the tryptophan pathway in the response variation of patients treated with sertraline, another selective serotonin reuptake inhibitor.^16, 17^ Furthermore, metabolomics approaches have been used to map the global effects of antipsychotics on metabolism,^16, 18, 19^ and in the case of first-episode neuroleptic-naive patients with schizophrenia, changes in purine and monoamine neurotransmitters, and the lipidome have been identified.^20^

Here, to our knowledge, we provide the first detailed metabolomics mapping and identify potential mechanisms of action, and biological pathways impacted by ketamine and esketamine by targeted and untargeted metabolomics platforms. Such platforms complement each other by allowing very precise quantitative analyses of a range of predefined metabolites. Gas chromatography–time-of-flight mass spectrometry (GC-TOF) is an untargeted technology for molecules <650 Da. Data are screened against the massive BinBase database that currently lists about 7000 unique compounds from over 150 000 samples run over the past 10 years. Most of these compounds are unknown, whereas ~1000 have been identified by the Fiehnlib or the NIST14 mass spectral libraries. Both known and unknown compounds are semi-quantified by relative peak heights and are used for generic novel hypotheses about metabolic regulation and finding novel biomarkers. In comparison, the Biocrates p180 platform complements this approach by targeting up to 188 endogenous metabolites from 5 different classes (acylcarnitines, amino acids, hexoses, phospholipids/sphingolipids and biogenic amines) by LC–MS/MS. The Biocrates p180 platform uses internal standards for absolute quantifications, making results comparable across studies and publications. The Biocrates p180 kit focuses on complex lipids (phospholipids, acylcarnitines and sphingolipids). The only partial overlap with the GC-TOF MS platform is for amino acids, for which the Biocrates p180 kit delivers more accurate quantifications than the untargeted screening approach. We compare and contrast metabolic signatures for the two enantiomers using targeted and non-targeted approaches measuring >400 metabolites on two metabolomics platforms in subjects with treatment-refractory MDD who received intravenous esketamine (*n*=20) or ketamine (*n*=33). We find novel metabolite signatures of ketamine and esketamine exposure and novel metabolite signatures of changes associated with decreased Montgomery-Åsberg Depression Rating Scale (MADRS).

## Materials and methods

### Subjects

For both ketamine and esketamine studies, the participants included men and women, 18–64 years old, who met Diagnostic and Statistical Manual of Mental Disorders, Fourth Edition, Text Revision (DSM-IV-TR)^21^ diagnostic criteria for recurrent MDD without psychotic features, based upon clinical assessment and the Mini International Neuropsychiatric Interview.^22^ Participants were required to have had an inadequate response to at least one antidepressant drug in their current depressive episode as well as an inadequate response to at least one other antidepressant either in their current or previous depressive episode, as assessed by the Massachusetts General Hospital-Antidepressant Treatment Response Questionnaire (MGH-ATRQ).^23^ At screening and on Day-1, patients had to have a total score ⩾34 on the Inventory of Depressive Symptomatology-Clinician rated, 30-item (IDS-C30).^24^ Additional participant information and exclusion criteria are available in the Supplementary Material.

The protocol and informed consent documents were approved by independent ethics committees or institutional review boards. Written informed consent was obtained from all participants.

The primary clinical endpoint was assessed using the change from baseline in the MADRS^25^ total score in the double-blind (DB) phase (see below). A 7-day recall period was used for the measurement of MADRS at baseline; whereas, a 24-h recall period was used for measurements at other time points.

### Study design and drug administration

In both studies, the medication was administered by continuous IV infusion using an electronic infusion pump managed by a physician/anesthesiologist experienced with ventilation management. Patients fasted overnight ⩾8 h before drug administration, until 2 h after the start of infusion, and the plasma sample used for the metabolomics analyses was obtained under fasting conditions.

Patients continued any antidepressant medications they were receiving at screening at the same doses throughout the study. An additional entrance criterion for only the ketamine trial was that independent SAFER raters from the Massachusetts General Hospital verified that all randomized patients met the SAFER criteria (defined as State versus trait, Assessability, Face validity, Ecological validity, and Rule of three Ps (pervasive, persistent, and pathological), had TRD according to the MGH-ATRQ, and had the IDS-C30 total score ⩾34 between the screening and the baseline visit.

Exclusion criteria for both studies included any primary DSM-IVTR diagnosis of panic disorder, obsessive compulsive disorder, posttraumatic stress disorder, anorexia nervosa, or bulimia nervosa; prior history or current diagnosis of psychotic disorder, bipolar disorder, mental retardation, or borderline personality disorders, mood disorder with postpartum onset, or somatoform disorders. Patients also were excluded if they had been hospitalized due to suicidal or homicidal ideation within the past 12 months, met criteria for substance abuse or dependence within 1 year prior (other than nicotine), or had a history of previous nonresponse to ketamine/esketamine.

#### Esketamine study

This DB, double-randomization, placebo-controlled, multi-center study comprised three phases: screening (up to 2 weeks), DB treatment (Days 1 to 7), and post-treatment (4 weeks, comprising an optional OL phase lasting up to 2 weeks and a follow-up phase) (https://clinicaltrials.gov/ct2/show/NCT01640080). On Day 1 (first dose) of the DB treatment phase, patients were randomized 1:1:1 to receive an IV infusion of 0.20 or 0.40 mg kg^−1^ esketamine or placebo (0.9% saline solution) over 40 min. Details of the randomization, blinding and rating procedures appear in Singh *et al.*^26^ On Day 4 (second dose) of the DB treatment phase, responders received the same treatment as Day 1. For non-responders the following rules were applied: (1) patients who received placebo on Day 1 were re-randomized 1:1 to IV esketamine 0.20 or 0.40 mg/kg; and (2) patients who received esketamine 0.20 or 0.40 mg/kg on Day 1 received esketamine 0.40 mg/kg on Day 4. The plasma samples were obtained 2 h from the first esketamine or placebo infusion and 2 h from the second esketamine infusion performed 3 days later.

#### Ketamine study

This was a randomized, DB, placebo-controlled, parallel-group, phase 2 study conducted at 14 sites in the USA that consisted of 4 phases: an up to 4-week screening phase, a 4-week DB treatment phase (Day 1 to Day 29), an optional 2-week open-label treatment phase, and an up to 3-week ketamine-free follow-up phase (https://clinicaltrials.gov/ct2/show/NCT01627782). During the DB treatment phase, patients were randomized (1:1:1:1) to one of four treatment groups: intravenous ketamine (0.5 mg kg^−1^) two or three times weekly or intravenous placebo (0.9% sodium chloride) two or three times weekly, administered over 40 min. The plasma sample used for the metabolomics assay was obtained 2 h from the first ketamine or placebo infusion.

#### Clinical phenotype evaluation

For each individual treated with esketamine, the percent change in MADRS score for the esketamine-treated group was determined as:


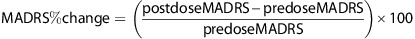


For individuals treated with esketamine, the postdose MADRS was assessed 2 h following esketamine administration.

For individuals treated with ketamine, the post-treatment MADRS assessment recorded closest to the administration of ketamine was used as the postdose MADRS value and was obtained approximately 2 to 4 days (x̄: 2.63 days, s=1.37 days) following ketamine treatment. The change in MADRS was calculated according to the above equation.

### Metabolite profiling

GC-TOF—study design information was entered into the miniX database (a simplified version of the SetupX database).^27^ All plasma samples were aliquoted and stored at −80 °C until use, at which point 30 μl of each sample was thawed, extracted and derivatized.^28^ All metabolites were measured as peak height. A total of 288 metabolites were measured (128 known and 160 unknown metabolites). GC-TOF MS data acquisition and processing were conducted as previously described.^29^ Additional information regarding the GC-TOF methods can be found in the Supplementary Material.

Biocrates P180—the Biocrates AbsoluteIDQ p180 kit assay (Innsbruck, Austria) was used for the quantification of amino acids, acylcarnitines, sphingomyelins, phosphatidylcholines, hexoses and biogenic amines. Additional information regarding the Biocrates p180 methods can be found in the Supplementary Material.

### Metabolite data processing

All the data analysis described below was performed using the statistical programming language, R.^30^ Additional details about metabolite data normalization, data processing, subject and metabolite outlier curation can be found in the Supplementary Material.

### Signature of drug exposure

The data were initially filtered to include only Day-1 samples. Samples were then split into pre-treatment and post-treatment groups. Each metabolite was tested to determine whether the change from pre- to post exposure was significantly different using a Wilcoxon signed-rank test for each drug exposure. Adjustments for multiple comparisons were made using a false-discovery rate approach.^31^

For the purposes of replication, the esketamine data were filtered to include only Day 4 samples. Metabolites that were significantly associated with esketamine treatment at Day 1 (*q*<0.25) then were tested for association using Day-4 samples. Metabolites were tested using Wilcoxon signed-rank test and considered to be statistically significant with a nominal *P*<0.05.

### Signature of drug response

In order to detect either pre-treatment values or changes in metabolites that associated with changes in MADRS scores, the data were filtered to include only Day-1 samples and were split into pre-treatment and post-treatment groups. Available covariates were tested for significance with the MADRS scores; however, none were statistically significantly (*q*<0.25). Additional information regarding the covariate selection can be found in the Supplementary Material.

Each pre-treatment metabolite level and change in metabolite level was tested for association with change in MADRS score using a linear regression model, and adjustments for multiple comparisons were performed using a false-discovery rate approach.^31^ Methods for performing the hierarchical clustering can be found in the Supplementary Materials.

## Results

### Response to ketamine and esketamine

Cohort demographics and clinical characteristics are presented in Table 1. For patients treated either with esketamine or ketamine the MADRS score was obtained prior to treatment and ~2h and 2-day post treatment, respectively (Supplementary Table 1). The mean absolute change in MADRS for the 33 subjects treated with ketamine was −10.48 (−26.73 to 5.76; 95% confidence interval (CI)) and a mean % change of −29.84% (−74.72 to 15.04; 95% CI). The mean absolute change in MADRS for the 20 subjects treated with esketamine was −16.05 (−34.28 to 2.08; 95% CI) and a mean % change of −47.73% (−99.37 to 3.91; 95% CI).

### Metabolite signature of ketamine exposure

**Figure 1:**
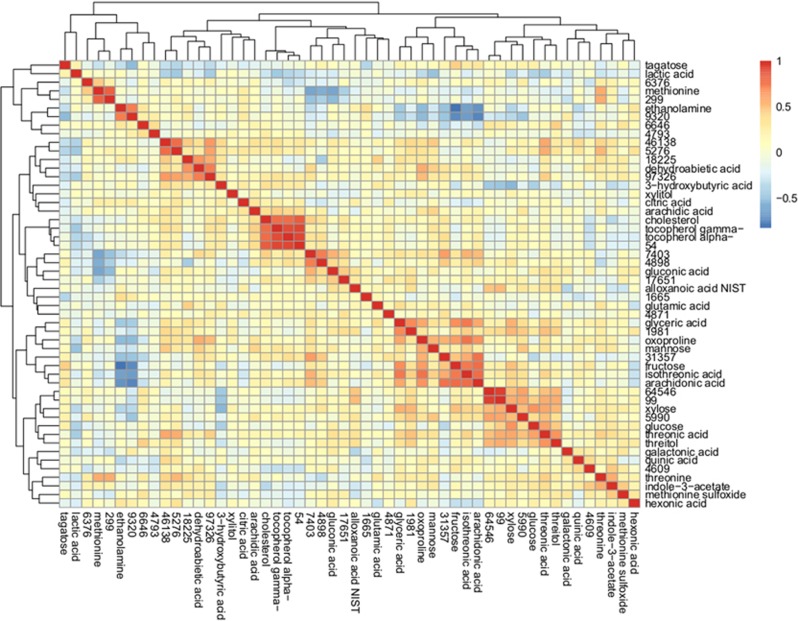
Hierarchical clustering of GC-TOF metabolites upon exposure to ketamine (*q*<0.25). Colors represent positively correlated (red) to negatively correlated (blue) metabolites. Some unknown metabolites are highly correlated with known metabolites, which may provide insight into their underlying function such as, methionine and 299, and also cholesterol, gamma-tocopherol, alpha-tocopherol and 54. GC-TOF, gas chromatography–time-of-flight mass spectrometry.

A total of 52 out of 288 metabolites on the GC-TOF platform were significantly altered upon treatment with ketamine (Table 2). Thirty one of these metabolites are known (for example, indole-3-acetate, 3-hydroxybutyric acid, arachidonic acid, lactic acid, methionine, mannose, fructose, gluconic acid, glyceric acid, isothreonic acid glutamic acid), and 21 are currently unknown metabolites (Table 2). As expected, hierarchical clustering revealed that gamma-tocopherol and alpha-tocopherol are positively correlated with each other, and with cholesterol (Figure 1). Unknown metabolite-9320 and ethanolamine were negatively correlated with arachidonic acid, isothreonic acid and fructose (Figure 1). Seven out of 188 metabolites on the Biocrates P180 platform were significantly altered upon treatment with ketamine (Table 2; *q*<0.25). Five of the seven metabolites significantly altered were acylcarnitines. Clustering analysis did not reveal any highly correlated metabolites (Supplementary Figure 3). No metabolite changed significantly in the placebo arm on either metabolomics platform (*q*<0.25).

### Metabolite signature of esketamine exposure

Six metabolites on the Biocrates p180 platform were significantly altered upon treatment with esketamine (hydroxybutyrylcarnitine, acetylcarnitine, hexose, isovalerylcarnitine/2-methylbutyrylcarnitine/valerylcarnitine and arginine) (Table 2; *q*<0.25). Interestingly, on the GC-TOF platform, unknown metabolite-18225 was significantly decreased with esketamine treatment (*q*=8.95x10^-4^) and was replicated with Day 4 data (*P*=1.19 × 10^−5^). This metabolite was significantly increased with exposure to ketamine (*q*=0.077). Indole-3-lactate and indole-3-acetate, both tryptophan metabolites, were decreased at Day 1 (*q*=0.08) and replicated in Day 4 (*P*=0.0012 and *P*=1.67 × 10^−6^, respectively). Clustering analysis revealed a low correlation with lyxitol and threonine (Supplementary Figure 4). No metabolite changed significantly in the placebo arm on either metabolomics platform (*q*<0.25).

### Metabolite signatures of response to treatment

No baseline metabolite significantly associated with response to treatment in subjects treated with ketamine, esketamine or placebo on either the GC-TOF or Biocrates P180 platform (*q*<0.25). In addition, no metabolite change significantly associated with response to treatment with esketamine on either platform. However, the metabolite changes from baseline to post-treatment for 65 metabolites were significantly associated with response to treatment in subjects treated with ketamine (Table 3). Many of the metabolites that were significantly altered were phosphatidylcholines, sphingomyelins or acylcarnitines. Ornithine and citrulline, intermediates of urea cycle, were negatively associated with MADRS change, meaning that as MADRS decreased (improved treatment response) these metabolites increased. In addition, the ratio of tryptophan:kynurenine was negatively associated with the % change in MADRS, indicating that the amount of tryptophan was increased relative to kynurenine in subjects with a greater percentage decrease in MADRS (*q*=0.23). Uric acid was the only metabolite that was significantly altered in response to placebo in the ketamine trial (*q*<0.25).

**Figure 2:**
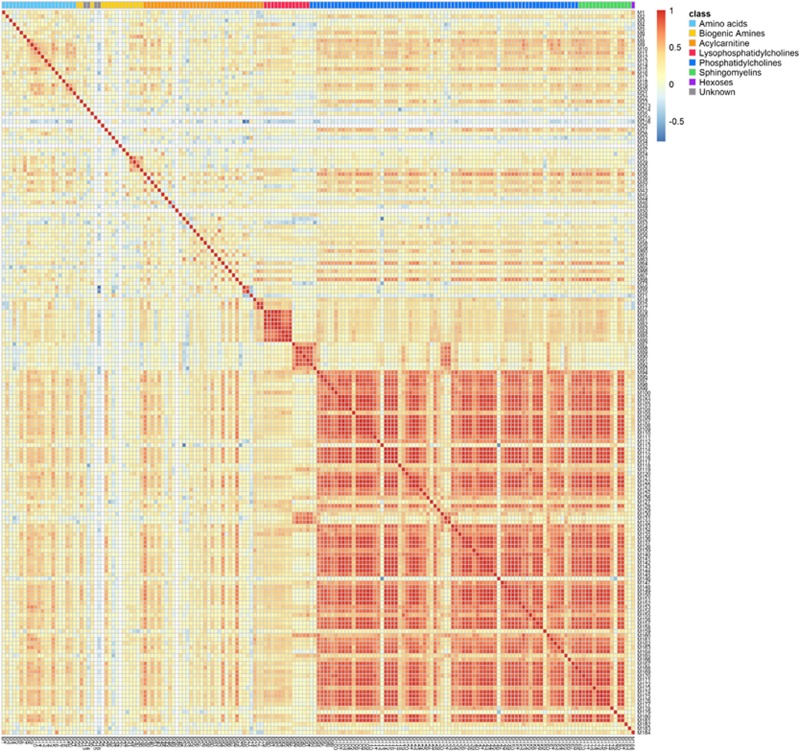
Heatmap displaying correlations of the changing metabolites from pre-treatment to post-treatment ketamine on the Biocrates platform. Distinct clusters of phosphatidylcholine and sphyngomyelin classes of metabolites show highly correlated changes from pre- to post-treatment ketamine.

The changes observed in phosphatidylcholines and sphingomyelins were highly correlated (Figure 2). PCA was performed with the annotated phosphatidylcholines (Supplementary Figure 5). The PCA shows that although there is not a clear separation between phosphatidylcholines and the other metabolites, there are two visually identifiable clusters indicating that the largest degree of variation is attributable to phosphatidylcholines compared to other classes of metabolites.

## Discussion

The data presented here show significant metabolite changes that are detectable in blood within 2 h of ketamine or esketamine treatment, and correlate with the antidepressant effect of ketamine ~2 days post-treatment. In each drug treatment group, esketamine was present as the most potent enantiomer for NMDA receptor antagonist effects. However, one treatment group received only the *S*-enantiomer, esketamine, whereas the other treatment group received the racemic mixture of ketamine. Thus, the results obtained in the two treatment groups provide complementary information, which may provide insight into both acute and persistent effects following drug exposure. Of the significant metabolite changes, the most notable changes suggest effects on the neurotransmitter-glutamic acid (glutamate), urea cycle and tryptophan metabolism. Altogether with the previously reported effects of ketamine treatment on energy metabolism and vascular function, the data presented here begin to establish a link between the systemic responses to ketamine treatment with the effect of ketamine/esketamine on depression symptoms.

Glutamic acid levels are increased 2 h following ketamine exposure (Table 2). The impact of ketamine administration on acute glutamate levels has been well documented in rodents and has shown that 5HT1B receptor activity requires activation of the glutamatergic AMPA receptor.^8, 32, 33^ Ketamine is known to block the glutamatergic NMDA receptor, thus the possible effect of increased glutamate levels could shift glutamatergic signaling from NMDA receptor to AMPA receptor to enhance the 5HT1B receptor activity that is hypothesized to be required for antidepressant effects.^34^ However, given that the reported effects of ketamine on glutamate in rodent models are very short lived, here using a new technique we were able to find a peripheral signature, which might still reflect this phenomenon well beyond the initial minutes after the administration. Recent data also suggest that downstream metabolites of ketamine may also be triggering downstream effects linked with antidepressant efficacy, such as activation of the mTOR pathway.^35^

Treatment with either compound, ketamine or esketamine, resulted in decreased tryptophan metabolites (indole-3-lactate and indole-3-acetate). In contrast, tryptophan and methionine levels were significantly decreased to one drug but not the other (Table 2), potentially reflecting differences in the effect of the compound. Furthermore, the effect on indole-3-lactate and indole-3-acetate seen in the first esketamine administration (Day 1) replicated in the second esketamine administration (Day 4; *P*<0.01), pointing toward a potential role for the gut microbiome (Table 2).^36, 37^ Acute pharmacokinetics data show comparable *C*_max_ between Day 1 and Day 4, indicating that the findings are not likely accounted by pharmacokinetic differences upon repeated administrations of esketamine.

In addition, unknown metabolite 18 225 was significantly increased with ketamine treatment and significantly decreased with esketamine treatment (Day 4 replication *P*=1.19 × 10^−5^). Interestingly, 18 225 has a mass-to-charge ratio of 179, similar to tyrosine, is aromatic and likely contains a nitrogen. Improving our understanding of unknown metabolites will be an important aspect of advancing metabolomics, as it promises new biological insights and may shed light on important aspects of drug action. Elucidating the structure of unknown metabolites remains a key bottleneck in metabolomics and structurally identifying these metabolites will require significant resources.

Previous work by Villaseñor *et al.*^38^ profiled changes in plasma metabolites in 22 patients with treatment-resistant bipolar disorder administered ketamine treatment, rather than unipolar patients presented here. Subjects in the study by Villaseñor *et al.* were also on valproate or lithium and were dichotomized into either ketamine responders or non-responders based on a 50% change in MADRS.^38^ Increased pre-treatment phospholipids (for example, lysophosphatidylcholines and lysophosphatidylethanolamines) were detected in individuals that responded to ketamine within 6 h versus those that did not respond. Despite differences in the study design, we also show that the change in many phosphotidylcholines and phosphoethanolamines 2 h post ketamine treatment were inversely associated with the % change in MADRS ~2 days post-treatment with ketamine (Table 3). Therefore, the concentrations of these metabolites increased in patients that experienced a larger reduction in depression symptoms relative to patients with a more modest response. Phosphatidylcholine is a major component of cell membranes, and these findings support evidence that ketamine increases synaptogenesis in the medial prefrontal cortex and hippocampus effects in preclinical rodent depression models.^10, 12^ These results are consistent with the hypothesis that the synthesis of these cell membrane components is greater in the patients whose depression severity is decreasing. Other studies have reported differences in fatty acid levels in MDD patients.^39^ An alternative explanation for this observation in response to ketamine may be owing to mild effects of ketamine and esketamine on renal filtration of lipids. Ketamine/esketamine are known NMDA receptor antagonists and NMDA receptor function in kidney has been shown to be required for glomerular filtration.^40^ Moreover, this association was observed with increasing clinical improvement to ketamine treatment, indicating potential differences in systemic NMDA antagonism in responders versus non-responders as one possible explanation for the increased phospholipid concentrations.

Finally, the data presented here suggest the possibility of increased metabolism of both dopamine and serotonin. Lindefors *et al.*^41^ and Moghaddam *et al.*^8^ showed dopamine is released following ketamine treatment in rat. Our data show that tyrosine, the amino-acid precursor to dopamine, was decreased 2 h following esketamine administration (Table 2). Dopamine activity is known to increase vascular tone and heart rate, and to block glucose dependent insulin release.^42, 43^ Altogether, increased dopamine activity following ketamine administration could account for the increased blood glucose levels (Table 2). Serotonin signaling through the 5HT1B receptor has also been recently identified as being required for the antidepressant effects of ketamine in macaques.^44^ Tryptophan, the amino-acid precursor to serotonin, and tryptophan metabolites, indole-3-acetate and indole-3-lactate, were decreased 2 h following esketamine treatment (Table 2). This is suggestive of a potential shift towards tryptophan metabolism towards serotonin, and away from indole-3-acetate and indole-3-lactate (Supplementary Figure 6). However, neither dopamine nor serotonin levels were significantly changed by ketamine administration, which may be owing to the limited sample size of the study or to the timing of the metabolomic assay relative to drug administration. Dopamine and serotonin signal through G-protein coupled receptors, which are internalized from the cell surface upon their activation. Therefore, given that dopamine and serotonin concentrations are known to increase following ketamine exposure in model organisms,^41, 45^ and their amine precursors (tryptophan and tyrosine) are decreased 2 h post-esketamine administration, it is possible that their increases were undetectable here owing to consumption at the cellular level owing to G-protein coupled receptors internalization,^46^ or were impacted by the gut microbiome as indicated based on effects of indole-containing metabolites (that is, indole-3-acetate and indole-3-lactate).^36, 37^ Another possibility is that the change in dopamine and serotonin levels preceded the timing of our measurements, and that the effect on precursor pools simply persisted beyond the time window when the elevation in the primary neurotransmitter levels was detectable; however, given that these changes occurred within 2 h it is not clear whether or not this is a plausible explanation.

As with any study, there are limitations that need to be considered. First, drug groups differ in the presence of *R*-ketamine, as one study was conducted with esketamine and the other with racemic ketamine However, pharmacokinetics data show that the observed *C*_max_ of 0.5 mg kg^−1^ racemic ketamine was comparable to the observed *C*_max_ of esketamine in the 0.4 mg kg^−1^ dose group based on the assumption that esketamine is ~3 × more potent than R-ketamine.^47, 48, 49^ The mean *C*_max_ of 0.2 mg kg^−1^ esketamine treatment was slightly lower than the *C*_max_ of 0.5 mg kg^−1^ racemic ketamine, but comparable efficacy was observed in both the 0.2 and 0.4 mg kg^−1^ esketamine groups. Another difference between the two studies concerns the timing of the post-treatment MADRS assessment (2 h versus 2 days). However, the MADRS scores at 2 h and 2 days are highly correlated for either drug, so this difference should not significantly limit the interpretation of the findings. Third, each group evaluated is from a cross-sectional sampling, so the data originate from a single point in time. In addition, subjects were receiving stable treatment with ineffective antidepressant therapies, which may impact baseline metabolite levels; however, the paired design of the study helps to mitigate confounding variables, and the lack of findings in the placebo group support this. Finally, the limited sample size, in combination with the large number of variables tested, limits the power of the current study. We reduced the likelihood of Type I error (false positives) using the false-discovery rate correction for multiple testing, but the risk of Type II error (false negatives) remained high. Importantly, except for change in uric acid in the subjects administered placebo, no other significant effects were observed in any tests conducted using the data from subjects administered placebo; therefore, lending additional support to suggest that the ketamine or esketamine metabolite effects were not the result of any confounding factors.

The data presented here, in tandem with previously published results, suggest that ketamine/esketamine administration could simultaneously facilitate increases in both glycolysis and oxidative phosphorylation via increased neurotransmitter metabolism, resulting in a rapid decrease in depression within 2 h of exposure. We consider all of the findings presented here as hypothesis generating in nature, and acknowledge that extensive additional studies are required to test all viable hypotheses in order to fully elucidate the mechanism by which ketamine/esketamine rapidly alleviates depressive symptoms. However, the analysis presented here represents, to our knowledge, the first large-scale, non-targeted, metabolomics analysis of both ketamine and esketamine in patients with MDD. Future analyses testing the functional nature of these changes are currently being implemented and the present study presents important findings supporting the therapeutic mechanism of ketamine and esketamine.

## Figures and Tables

**Table 1:** Cohort demographics

	*Ketamine trial*		*Esketamine trial*	
	*Ketamine (*n=*33)*	*Placebo (*n=*12)*	*Esketamine (*n=*20)*	*Placebo (*n=*10)*
Age—mean (s.d.)	44.55 (10.35)	45.17 (8.94)	43.1 (12.20)	42.70 (10.89)
Gender (% female)	69.69%	67.67%	60%	60%
Baseline MADRS—mean (s.d.)	34.48 (5.21)	34.33 (2.93)	33.45 (4.82)	33.90 (4.15)
Post-treatment MADRS—mean (s.d.)	24.00 (7.87)	31.73 (5.24)	17.40 (8.62)	28.80 (4.66)
MADRS change—mean (s.d.)	−10.48 (8.12)	−2.64 (4.82)	−16.05 (9.06)	−5.1 (6.08)
MADRS change %—mean (s.d.)	−29.84% (22.44)	−7.56 (12.98)	−47.73 (25.82)	−14.10 (16.88)
				
*Concomitant medications*				
Selective serotonin reuptake inhibitors, *n* (%)	26 (78.79%)	9 (75%)	5 (25%)	3 (30%)
Beta-blocking agents, non-selective, *n* (%)	0 (0%)	0 (0%)	0 (0%)	0 (0%)
Barbituates and derivatives, *n* (%)	0 (0%)	0 (0%)	1 (5%)	0 (0%)
Non-steroidal anti-inflammatory drugs, *n* (%)	0 (0%)	0 (0%)	0 (0%)	0 (0%)
Benzodiazepine derivatives, *n* (%)	0 (0%)	0 (0%)	7 (35%)	4 (40%)
Monoamine reuptake inhibitors, non-selective, *n* (%)	1 (3.03%)	1 (8.33%)	3 (15%)	5 (50%)
Dopamine agonists, *n* (%)	0 (0%)	0 (0%)	1 (5%)	0 (0%)
Other antidepressants, *n* (%)	12 (36.36%)	8 (66.67%)	15 (75%)	5 (50%)
Diazepines, oxazepines, thiazepines and oxepines, *n* (%)	0 (0%)	0 (0%)	3 (15%)	2 (20%)
Monoamine oxidase inhibitors, non-selective, *n* (%)	1 (3.03%)	0 (0%)	1 (5%)	0 (0%)

Abbreviation: MADRS, Montgomery-Åsberg Depression Rating Scale.

**Table 2:** Metabolite Signature of ketamine and esketamine exposure

*Treatment*	*Metabolite platform*	*Metabolite*	N	*Pre-treatment mean*	*Post-treatment mean*	*Delta*	*Wilcoxon* P*-value*	*Wilcoxon* q*-value*	*Day 4 replication* P*-value*
Ketamine	GC-TOF	Mannose	33	3.02	3.06	0.04	8.08E−06	0.002	
		Fructose	33	2.91	3.20	0.30	1.44E−05	0.002	
		Gluconic acid	33	2.96	3.12	0.15	7.54E−05	0.007	
		Glyceric acid	33	2.98	3.07	0.09	1.73E−04	0.013	
		Isothreonic acid	33	2.98	3.06	0.08	4.75E−04	0.027	
		Galactonic acid	33	2.95	3.15	0.20	6.51E−04	0.027	
		5276	33	3.58	3.63	0.04	9.52E−04	0.027	
		Glutamic acid	33	2.99	3.10	0.11	9.52E−04	0.027	
		9320	33	3.35	3.06	−0.29	0.001	0.027	
		Oxoproline	33	3.02	3.04	0.03	0.001	0.027	
		97326	33	2.41	2.55	0.14	0.001	0.027	
		Tocopherol gamma-	33	3.10	3.00	−0.10	0.002	0.027	
		6376	33	2.37	2.27	−0.10	0.002	0.027	
		Glucose	33	3.02	3.05	0.03	0.002	0.027	
		Xylitol	33	2.97	3.05	0.09	0.002	0.027	
		64546	33	3.74	3.82	0.08	0.002	0.027	
		Dehydroabietic acid	33	2.96	3.09	0.13	0.002	0.027	
		Quinic acid	33	3.12	3.00	−0.12	0.002	0.027	
		46138	33	3.59	3.62	0.04	0.003	0.039	
		Methionine sulfoxide	33	3.01	3.08	0.07	0.003	0.045	
		Citric acid	33	3.01	3.04	0.03	0.004	0.052	
		99	33	3.74	3.82	0.08	0.005	0.060	
		18225	33	3.12	3.23	0.11	0.006	0.078	
		Indole-3-acetate	33	3.07	3.01	−0.06	0.007	0.084	
		3-Hydroxybutyric acid	33	2.92	3.07	0.15	0.007	0.086	
		Xylose	33	2.98	3.08	0.09	0.008	0.087	
		4871	33	2.53	2.58	0.05	0.009	0.092	
		Arachidonic acid	33	2.98	3.12	0.14	0.010	0.102	
		Lactic acid	33	3.07	2.99	−0.08	0.010	0.104	
		1981	33	3.01	2.97	−0.04	0.011	0.106	
		1665	33	3.50	3.57	0.07	0.012	0.109	
		Tocopherol alpha-	33	3.05	2.98	−0.07	0.012	0.111	
		Methionine	33	3.08	2.97	−0.11	0.018	0.151	
		Alloxanoic acid NIST	33	3.13	2.95	−0.18	0.019	0.151	
		4793	33	2.05	2.17	0.13	0.019	0.151	
		Threitol	33	3.02	2.96	−0.06	0.019	0.151	
		7403	33	2.30	2.47	0.17	0.023	0.180	
		4609	33	2.32	2.36	0.04	0.024	0.184	
		5990	33	2.40	2.31	−0.09	0.027	0.193	
		Threonic acid	33	3.00	3.02	0.03	0.027	0.193	
		299	33	2.92	2.83	−0.09	0.031	0.212	
		54	33	3.37	3.31	−0.07	0.031	0.212	
		Cholesterol	33	3.04	3.01	−0.04	0.032	0.217	
		Ethanolamine	33	3.09	2.97	−0.12	0.034	0.217	
		4898	33	2.82	2.75	−0.08	0.034	0.217	
		Threonine	33	3.05	3.06	−0.01	0.035	0.223	
		17651	33	2.37	2.46	0.09	0.037	0.229	
		Hexonic acid	33	2.99	3.08	0.09	0.039	0.229	
		Arachidic acid	33	3.07	3.00	−0.07	0.039	0.229	
		Tagatose	33	3.01	3.10	0.09	0.042	0.241	
		31357	33	2.80	2.86	0.07	0.042	0.241	
		6646	33	2.66	2.69	0.03	0.044	0.247	
	Biocrates	Hydroxybutyrylcarnitine (malonylcarnitine) (C4-OH (C3-DC))	33	−0.10	0.10	0.20	4.36E−04	0.044	
		Acetylcarnitine (C2)	33	−0.32	0.40	0.72	5.57E-04	0.044	
		Hexose (H1)	33	−0.36	0.03	0.39-	0.001	0.078	
		Isovalerylcarnitine/2-methylbutyrylcarnitine/valerylcarnitine (C5)	33	0.21	−0.02	−0.23	0.004	0.153	
		Arginine (Arg)	33	−0.04	0.02	0.06	0.006	0.196	
		Butyrylcarnitine / Isobutyrylcarnitine (C4)	33	0.53	0.29	−0.24	0.011	0.249	
		Hexadecanoylcarnitine (=palmitoylcarnitine) (C16)	33	−0.24	0.08	0.32	0.011	0.249	
									
Esketamine	GC-TOF	18225	20	3.19	2.94	−0.25	3.81E−06	0.001	**1.19E**−**05**
		Indole-3-lactate	20	3.01	2.95	−0.06	0.001	0.087	**0.001**
		Threonine	20	3.03	3.00	−0.02	0.001	0.087	**0.031**
		Indole-3-acetate	20	3.08	2.97	−0.11	0.001	0.087	**1.67E**−**06**
		lyxitol	20	3.05	3.00	−0.05	0.004	0.177	**1.95E**−**04**
	Biocrates	Tryptophan (Trp)	19	0.73	−0.23	−0.95	3.36E−04	0.060	**0.178**
		Ornithine (Orn)	19	0.41	0.06	−0.34	0.003	0.184	**0.552**
		Alanine (Ala)	19	0.66	0.13	−0.53	0.005	0.184	**0.011**
		Propionylcarnitine (C3)	19	0.26	0.04	−0.23	0.005	0.184	**0.019**
		Tyrosine (Tyr)	19	0.39	−0.27	−0.66	0.006	0.184	**0.465**
		Butyrylcarnitine/isobutyrylcarnitine (C4)	19	0.01	−0.22	−0.23	0.006	0.184	**3.33E**−**06**

Abbreviation: GC-TOF, gas chromatography–time-of-flight. Bold entries represent *P*-values from day 4 replication.

**Table 3:** Biocrates changing metabolites signature of ketamine treatment response—KETIVTRD2002

*Biocrates code*	*Metabolite*	P-*value*	q*-value*	R^*2*^	*Direction of association with MADRS score, % change*
PC ae C34:0	Phosphatidylcholine with acyl-alkyl residue sum C34:0	0.0079	0.2213	0.1810	Negative
Orn	Ornithine	0.0092	0.2213	0.1737	Negative
C18:2	Octadecadienoylcarnitine (=linoleylcarnitine)	0.0139	0.2213	0.1535	Negative
C16	Hexadecanoylcarnitine (=palmitoylcarnitine)	0.0154	0.2213	0.1482	Negative
PC ae C36:3	Phosphatidylcholine with acyl-alkyl residue sum C36:3	0.0156	0.2213	0.1477	Negative
PC ae C38:3	Phosphatidylcholine with acyl-alkyl residue sum C38:3	0.0160	0.2213	0.1465	Negative
PC ae C44:5	Phosphatidylcholine with acyl-alkyl residue sum C44:5	0.0217	0.2213	0.1314	Negative
ADMA	Asymmetric dimethylarginine	0.0229	0.2213	0.1288	Negative
PC ae C32:1	Phosphatidylcholine with acyl-alkyl residue sum C32:1	0.0250	0.2213	0.1245	Negative
PC ae C40:4	Phosphatidylcholine with acyl-alkyl residue sum C40:4	0.0294	0.2213	0.1163	Negative
PC aa C42:5	Phosphatidylcholine with diacyl residue sum C42:5	0.0315	0.2213	0.1130	Negative
SM C18:1	Sphingomyelin with acyl residue sum C18:1	0.0346	0.2213	0.1082	Negative
C18:1	Octadecenoylcarnitine (=oleylcarnitine)	0.0364	0.2213	0.1057	Negative
PC ae C42:4	Phosphatidylcholine with acyl-alkyl residue sum C42:4	0.0368	0.2213	0.1051	Negative
SM C16:0	Sphingomyelin with acyl residue sum C16:0	0.0404	0.2213	0.1005	Negative
SM C16:1	Sphingomyelin with acyl residue sum C16:1	0.0430	0.2213	0.0974	Negative
SM (OH) C22:1	Hydroxysphingomyelin with acyl residue sum C22:1	0.0436	0.2213	0.0967	Negative
PC ae C38:5	Phosphatidylcholine with acyl-alkyl residue sum C38:5	0.0439	0.2213	0.0964	Negative
C5-DC (C6-OH)	Glutarylcarnitine (Hydroxyhexanoylcarnitine (=hydroxycaproylcarnitine))	0.0505	0.2213	0.0894	Negative
PC ae C40:6	Phosphatidylcholine with acyl-alkyl residue sum C40:6	0.0510	0.2213	0.0889	Negative
PC ae C36:4	Phosphatidylcholine with acyl-alkyl residue sum C36:4	0.0535	0.2213	0.0865	Negative
SM C24:0	Sphingomyelin with acyl residue sum C24:0	0.0577	0.2213	0.0827	Negative
PC ae C36:1	Phosphatidylcholine with acyl-alkyl residue sum C36:1	0.0596	0.2213	0.0810	Negative
PC ae C34:2	Phosphatidylcholine with acyl-alkyl residue sum C34:2	0.0599	0.2213	0.0808	Negative
PC ae C36:2	Phosphatidylcholine with acyl-alkyl residue sum C36:2	0.0600	0.2213	0.0807	Negative
C7-DC	Pimelylcarnitine	0.0603	0.2213	0.0805	Negative
PC aa C38:3	Phosphatidylcholine with diacyl residue sum C38:3	0.0613	0.2213	0.0797	Negative
SM C18:0	Sphingomyelin with acyl residue sum C18:0	0.0618	0.2213	0.0793	Negative
PC aa C40:5	Phosphatidylcholine with diacyl residue sum C40:5	0.0671	0.2213	0.0751	Negative
SM C24:1	Sphingomyelin with acyl residue sum C24:1	0.0674	0.2213	0.0750	Negative
PC ae C36:0	Phosphatidylcholine with acyl-alkyl residue sum C36:0	0.0690	0.2213	0.0738	Negative
SM (OH) C16:1	Hydroxysphingomyelin with acyl residue sum C16:1	0.0696	0.2213	0.0733	Negative
PC ae C34:3	Phosphatidylcholine with acyl-alkyl residue sum C34:3	0.0716	0.2213	0.0720	Negative
PC ae C38:4	Phosphatidylcholine with acyl-alkyl residue sum C38:4	0.0727	0.2213	0.0712	Negative
C16:1-OH	Hydroxyhexadecenoylcarnitine (=hydroxypalmitoleylcarnitine)	0.0728	0.2213	0.0711	Negative
PC ae C40:2	Phosphatidylcholine with acyl-alkyl residue sum C40:2	0.0738	0.2213	0.0704	Negative
PC ae C36:5	Phosphatidylcholine with acyl-alkyl residue sum C36:5	0.0755	0.2213	0.0693	Negative
Ala	Alanine	0.0764	0.2213	0.0687	Positive
PC ae C38:0	Phosphatidylcholine with acyl-alkyl residue sum C38:0	0.0858	0.2304	0.0629	Negative
SM (OH) C14:1	Hydroxysphingomyelin with acyl residue sum C14:1	0.0876	0.2304	0.0619	Negative
SM (OH) C22:2	Hydroxysphingomyelin with acyl residue sum C22:2	0.0880	0.2304	0.0617	Negative
PC aa C36:2	Phosphatidylcholine with diacyl residue sum C36:2	0.0913	0.2304	0.0598	Negative
PC aa C38:4	Phosphatidylcholine with diacyl residue sum C38:4	0.0917	0.2304	0.0597	Negative
C16:1	Hexadecenoylcarnitine (=palmitoleylcarnitine)	0.0983	0.2304	0.0562	Negative
PC aa C36:4	Phosphatidylcholine with diacyl residue sum C36:4	0.0995	0.2304	0.0556	Negative
PC ae C32:2	Phosphatidylcholine with acyl-alkyl residue sum C32:2	0.1021	0.2304	0.0543	Negative
C18	Octadecanoylcarnitine (=searylcarnitine)	0.1025	0.2304	0.0541	Negative
C14:1	Tetradecenoylcarnitine (=myristoleylcarnitine)	0.1035	0.2304	0.0536	Negative
PC ae C44:6	Phosphatidylcholine with acyl-alkyl residue sum C44:6	0.1059	0.2304	0.0525	Negative
PC aa C40:1	Phosphatidylcholine with diacyl residue sum C40:1	0.1074	0.2304	0.0518	Negative
PC ae C38:1	Phosphatidylcholine with acyl-alkyl residue sum C38:1	0.1090	0.2304	0.0511	Positive
PC ae C38:2	Phosphatidylcholine with acyl-alkyl residue sum C38:2	0.1111	0.2304	0.0501	Negative
PC aa C42:2	Phosphatidylcholine with diacyl residue sum C42:2	0.1125	0.2304	0.0495	Negative
PC ae C38:6	Phosphatidylcholine with acyl-alkyl residue sum C38:6	0.1171	0.2304	0.0476	Negative
Tryptophan_Kynurenine	Tryptophan : Kynurenine Ratio	0.1182	0.2304	0.0471	Negative
PC aa C42:0	Phosphatidylcholine with diacyl residue sum C42:0	0.1187	0.2304	0.0469	Negative
PC ae C34:1	Phosphatidylcholine with acyl-alkyl residue sum C34:1	0.1194	0.2304	0.0466	Negative
PC aa C36:3	Phosphatidylcholine with diacyl residue sum C36:3	0.1214	0.2304	0.0458	Negative
PC aa C36:1	Phosphatidylcholine with diacyl residue sum C36:1	0.1246	0.2325	0.0445	Negative
PC aa C34:4	Phosphatidylcholine with diacyl residue sum C34:4	0.1311	0.2399	0.0421	Negative
PC aa C34:1	Phosphatidylcholine with diacyl residue sum C34:1	0.1363	0.2399	0.0402	Negative
PC aa C36:5	Phosphatidylcholine with diacyl residue sum C36:5	0.1368	0.2399	0.0400	Negative
PC aa C38:5	Phosphatidylcholine with diacyl residue sum C38:5	0.1372	0.2399	0.0398	Negative
PC aa C38:1	Phosphatidylcholine with diacyl residue sum C38:1	0.1398	0.2405	0.0390	Negative
PC aa C42:6	Phosphatidylcholine with diacyl residue sum C42:6	0.1423	0.2412	0.0381	Negative

Abbreviation: MADRS, Montgomery-Åsberg Depression Rating Scale.

## Supplementary Material

Supplementary Informations
