# Supplementary material for: Metabolomic signatures of drug response phenotypes for ketamine and esketamine in subjects with refractory major depressive disorder: new mechanistic insights for rapid acting antidepressants
Source: Transl Psychiatry. 2016 Sep 20;6(9):e894–. doi: 10.1038/tp.2016.145 (PMC5048196; doi:10.1038/tp.2016.145)
Supplement: Supplementary Informations [file tp2016145x1.doc]

**Supplemental Information**

**Metabolomic Signatures of drug response phenotypes for ketamine and esketamine in subjects with refractory major depressive disorder - - New mechanistic insights for rapid acting antidepressants.**

Daniel M. Rotroff^1,2^, Daniel G. Corum^3^, Alison Motsinger-Reif^1,2^, Oliver Fiehn^5^, Nicole Bottrel^4^,Wayne C. Drevets^4^, Jaskaran Singh^4^, Giacomo Salvadore^4*^, Rima Kaddurah-Daouk^6,7*^

1. Department of Statistics, North Carolina State University, Raleigh NC
2. Bioinformatics Research Center, North Carolina State University, Raleigh NC
3. Department of Drug Discovery and Biomedical Sciences, Medical University of South Carolina, Charleston, SC
4. Janssen Research and Development, Titusville, NJ, USA
5. UC Davis Genome Center, University of California Davis, Davis, CA
6. Department of Psychiatry, Duke University Medical Center, Durham NC
7. Duke Institute for Brain Sciences, Duke University, Durham, NC, USA;

*Corresponding Author:

Rima Kaddurah-Daouk, Ph.D.

Box 3903

3552, Blue Zone, Duke South

Durham, NC 27710

Email: rima.kaddurahdaouk@duke.edu

*Giacomo Salvadore, M.D.,

Janssen Pharmaceuticals,

1125 Trenton Harbourton Road,

Titusville, NJ 08560, USA.
E-mail: gsalvado@its.jnj.com

*Subjects*

Patients continued any antidepressant medications they were receiving at screening at the same doses throughout the study. An additional entrance criterion for only the ketamine trial was that independent SAFER raters from the Massachusetts General Hospital verified that all randomized patients met the SAFER criteria (defined as State versus trait, Assessability, Face validity, Ecological validity, and Rule of three Ps (pervasive, persistent, and pathological), had TRD according to the MGH-ATRQ, and had the IDS-C30 total score ≥34 between the screening and the baseline visit.

Exclusion criteria for both studies included any *primary* DSM-IV-TR diagnosis of panic disorder, obsessive compulsive disorder, posttraumatic stress disorder, anorexia nervosa, or bulimia nervosa; prior history or current diagnosis of psychotic disorder, bipolar disorder, mental retardation, or borderline personality disorders, mood disorder with postpartum onset, or somatoform disorders. Patients also were excluded if they had been hospitalized due to suicidal or homicidal ideation within the past 12 months, met criteria for substance abuse or dependence within 1 year prior (other than nicotine), or had a history of previous non-response to ketamine/esketamine.

*Study Design and Drug Administration*

*Esketamine Study*:

On Day 4 (second dose) of the DB treatment phase, responders received the same treatment as Day 1. For non-responders the following rules were applied: (1) patients who received placebo on Day 1 were re-randomized 1:1 to IV esketamine 0.20 or 0.40 mg/kg; and (2) patients who received esketamine 0.20 or 0.40 mg/kg on Day 1 received esketamine 0.40 mg/kg on Day 4. Details of the randomization, blinding and rating procedures appear in Singh et al^1^.

*Metabolite Profiling*

GC-TOF: Study design information was entered into the miniX database (a simplified version of the SetupX database)^2^. All plasma samples were aliquoted and stored at −80°C until use, at which point 30 µl of each sample was thawed, extracted and derivatized^3^. Briefly, 30 µl aliquots were extracted with 1 ml of degassed acetonitrile:isopropanol:water (3:3:2) at −20°C, centrifuged, aliquoted into two portions and evaporated to complete dryness. Acetonitrile/water (1:1) was used to remove membrane lipids and triglycerides and the supernatant was again dried down. Internal standards C8–C30 FAMEs were added and the sample was derivatized using methoxyamine hydrochloride in pyridine and subsequently by MSTFA (Sigma-Aldrich) for trimethylsilylation of acidic protons. All metabolites were measured as peak height. A total of 288 metabolites were measured (128 known and 160 unknown metabolites). GC-TOF MS data acquisition and processing were conducted as previously described^4^.

Biocrates P180: The Biocrates AbsoluteIDQ® p180 Kit assay was used for the quantification of amino acids, acylcarnitines, sphingomyelins, phosphatidylcholines, hexoses, and biogenic amines. Frozen samples were sent to Biocrates Life Sciences AG (Innsbruck, Austria) for analysis. The fully automated assay was based on PITC (phenylisothiocyanate)-derivatization in the presence of internal standards followed by FIA-MS/MS (acylcarnitines, lipids, and hexose) and LC/MS (amino acids, biogenic amines) using an AB SCIEX 4000 QTrap® mass spectrometer (AB SCIEX, Darmstadt, Germany) with electrospray ionization. Accuracy of the measurements (determined with the accuracy of the calibrators) was in the normal range of the methods (deviations from target ≤ 20 %) for all analytes. Measurements for FIA-part of the AbsoluteIDQ® p180 kit assay have been batch corrected for analytes > LOD by using pooled plasma reference samples. The experimental metabolomics measurement technique is described in detail by patent US 2007/0004044^5^.

*Metabolite Data Processing*

All data analysis described below was performed using the statistical programming language, R^6^. Metabolite data on both the Biocrates and GC-TOF platforms was Log10 transformed. For the purposes of normalization, the data were analyzed separately by gender and drug treatment to determine whether the global metabolic profiles differed based on these parameters. Stratifications tested included male/female, ketamine/esketamine, and male+ketamine/female+ketamine/male+esketamine/female+esketamine. Differences were determined by both Student’s t-test and the non-parametric Mann-Whitney U test. This is an important step because if the metabolic profiles are different between subgroups then the normalization procedure must be performed on each subgroup independently. Significant differences were noted between all groups tested (p<0.05), so a form of vector normalization was performed on each subgroup, and then recombined, post normalization.

metabolite*_ij_*_,normalized_ = metabolite*_ij_*_, raw_ / mTIC*_j_* * mTIC_median_

mTIC is the sum of metabolite values across all subjects for a given metabolite.

Some metabolite values in the Biocrates data file were marked <LOD in some subjects. The value marked <LOD was replaced with ½ the actual LOD value. The data were stratified by male/female and ketamine/esketamine to determine whether metabolic profiles were different between subgroups. No difference in a global metabolic profile was statistically different between male/female or ketamine/esketamine as determined by Student’s t-test or the non-parametric Mann-Whitney U test (p<0.05). For this reason, all subgroups were combined for the purposes of normalization. Lastly, values were median centered and then scaled using the standard deviation of each metabolite.

There were no subjects removed due to missing values or outliers as determined by PCA (Supplementary Figures 1 and 2). Capric Acid on the GC-TOF platform and Putrescine, C3-OH, C3:1, C5:1, C12-DC, C16:2=OH, C18:1-OH, lysoPC a C14:0, PC aa C30:2 on the Biocrates platform showed no variation between subjects and were subsequently removed.

*Covariate Selection*

For the signature of response analysis, covariates (age, sex, ethnicity, country, BMI, arm, race, and all concomitant medications) were tested against the change in MADRS for association using a linear regression model. Categorical variables were dummy coded, and results were adjusted for multiple comparisons using a FDR approach^7^. However, using a statistical threshold of q < 0.25, no covariate was significantly associated with the MADRS scores, so the covariates tested were subsequently excluded from the linear regression models.

*Hierarchical Clustering*

For each signature of drug exposure and signature of drug response analysis, significant metabolites (*q*< 0.25) were clustered. Hierarchical clustering was performed using the pheatmap package^8^ in R^6^. Clustering was performed using Euclidean distance and Ward’s method.

*Discussion*

Arginine levels were increased following ketamine exposure, and arginine precursors, citrulline and ornithine, were significantly increased as the MADRS decreases in subjects treated with ketamine (Tables 2 and 3). These impacts on urea cycle metabolites further implicate the mitochondria. Recent studies have suggested that mitochondrial dysfunction may play an important role in the pathophysiology of MDD and bipolar disorder. Increases in mitochondrial DNA copy-number aberrations and deletions have been observed in patients with MDD and bipolar disorder^12,13^. Furthermore, patients with MDD have been shown to have increased mitochondrial oxidative damage relative to comparison groups^14,15^.

Endothelial nitric oxide synthase consumes arginine to produce nitric oxide (NO) in order to cause vasodilation^16^. Given the known, albeit short-lived, vasoconstrictive effects of ketamine administration, one explanation for increased arginine observed in our study is that arginine levels may be increased post-ketamine exposure due to a compensatory effect to balance the vasoconstrictive effect of ketamine. A recent study by Liebenberg et al. demonstrated that arginine pretreatment attenuated the antidepressant effect of ketamine in Flinders sensitive line rats^17^. Nitric oxide, similar to ketamine, is a known NMDA antagonist, and administration of nitrous oxide, which is converted to NO, has also been shown to rapidly decrease depression symptoms in individuals with MDD^18–21^. The role of NO and nitric oxide synthase isoforms in depression is complex and additional research is needed to fully characterize its role and potential therapeutic benefits^22^.


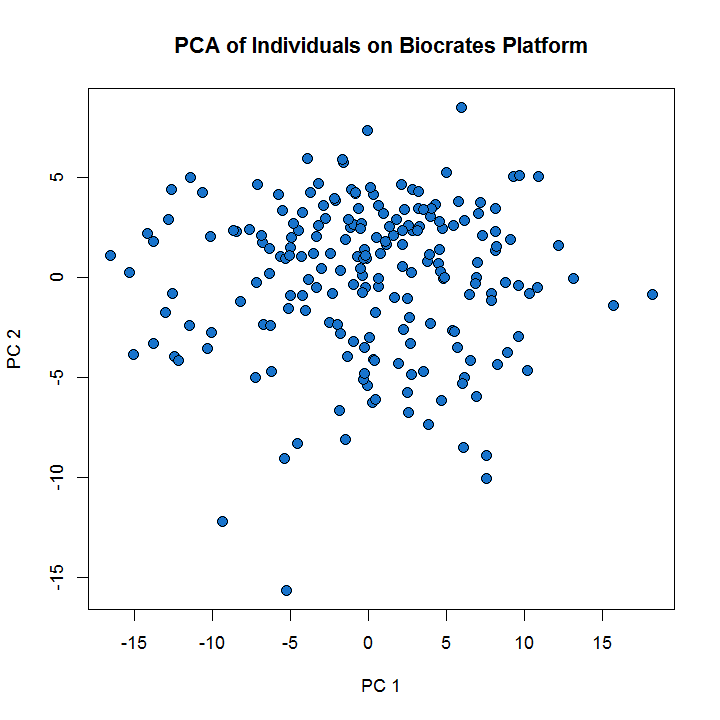


Supplemental Figure 1. Principal Component Analysis of Individuals based on Biocrates metabolite profiles.

Supplemental Figure 2. Principal Component Analysis of Individuals based on GC-TOF metabolite profiles.


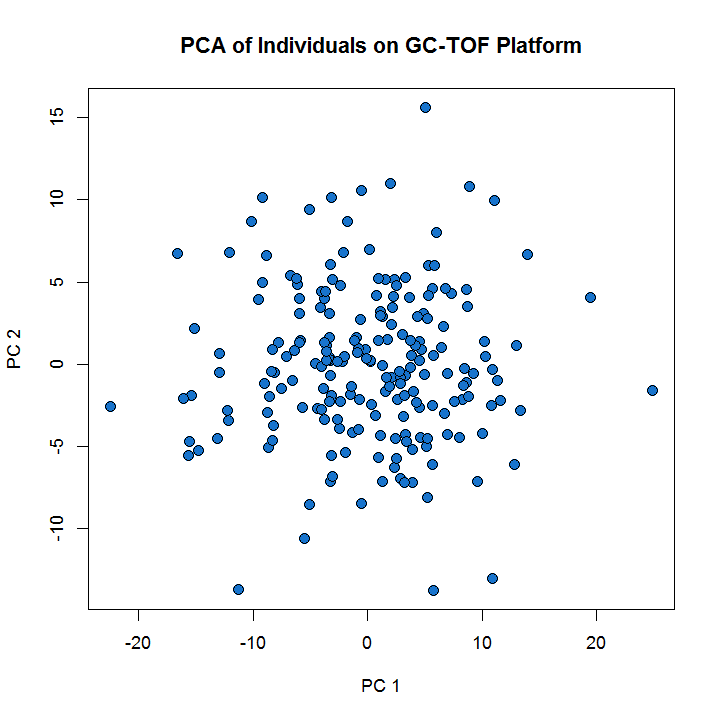


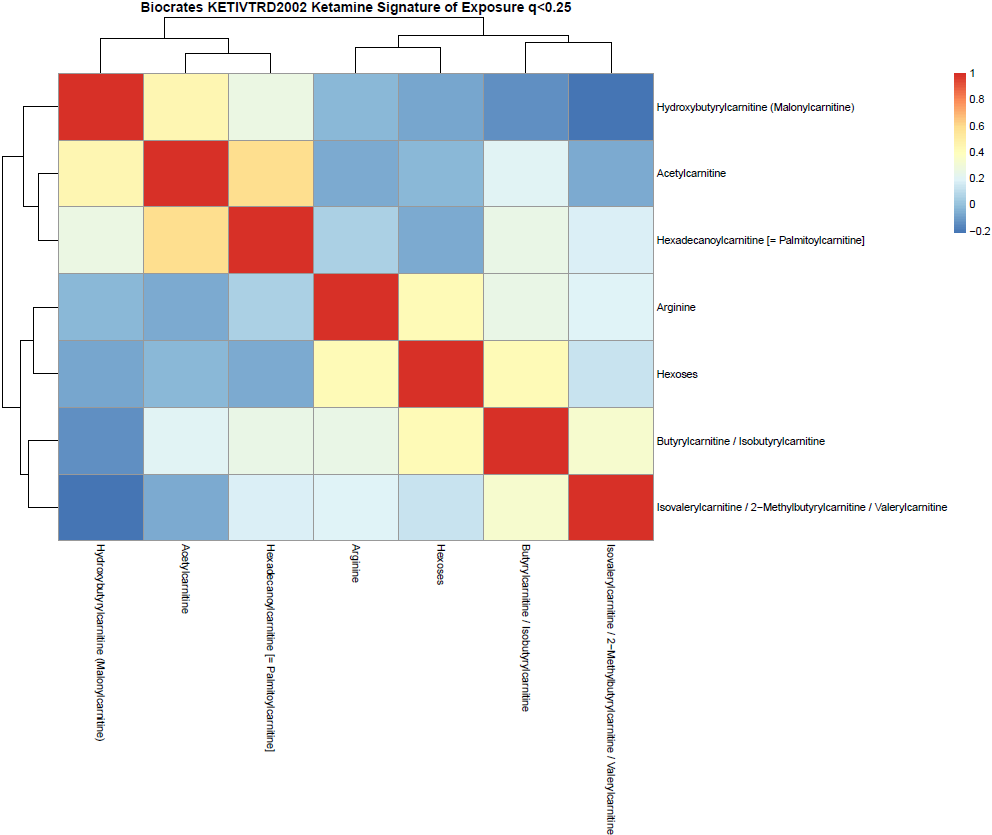


Supplemental Figure 3. Biocrates Ketamine Signature of Exposure (*q* < 0.25).


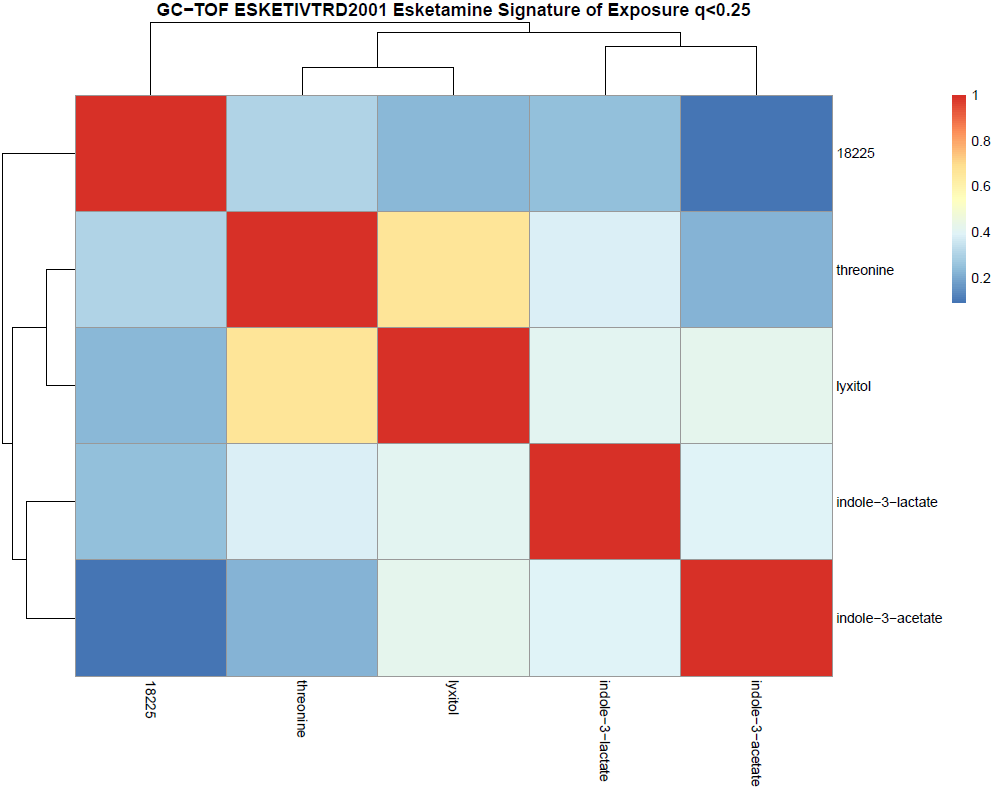
Supplemental Figure 4. GC-TOF Esketamine Signature of Exposure (*q* < 0.25).

**
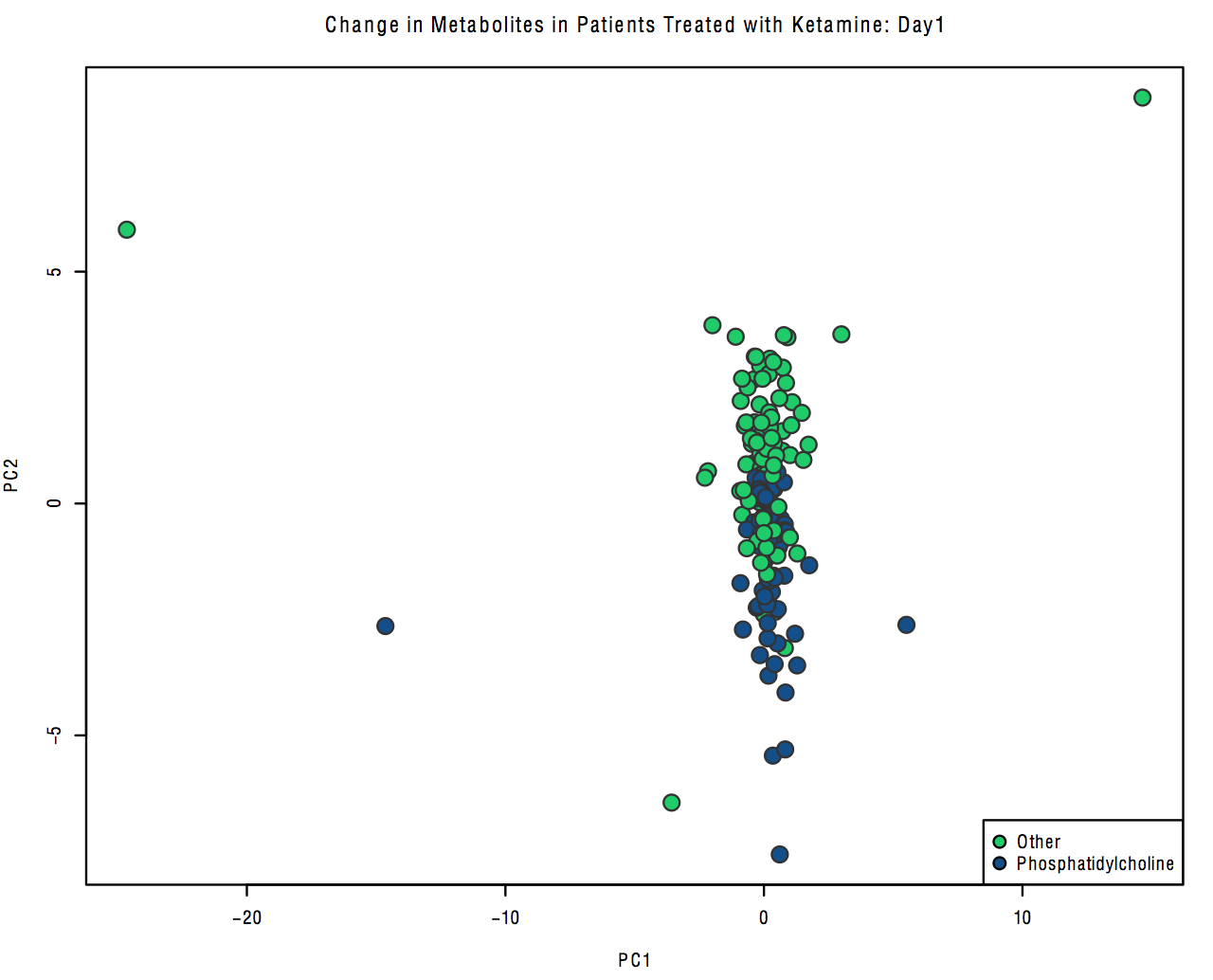
**

**Supplemental Figure 5.** Principal component analysis of metabolite changes in patients treated with ketamine at day 1 as measured by the Biocrates platform. Blue points represent phosphatidylcholine metabolites. Although, a distinct separation is not visible, phosphatidylcholine metabolites can be observed clustering together relative to the other metabolites measured by the Biocrates platform.


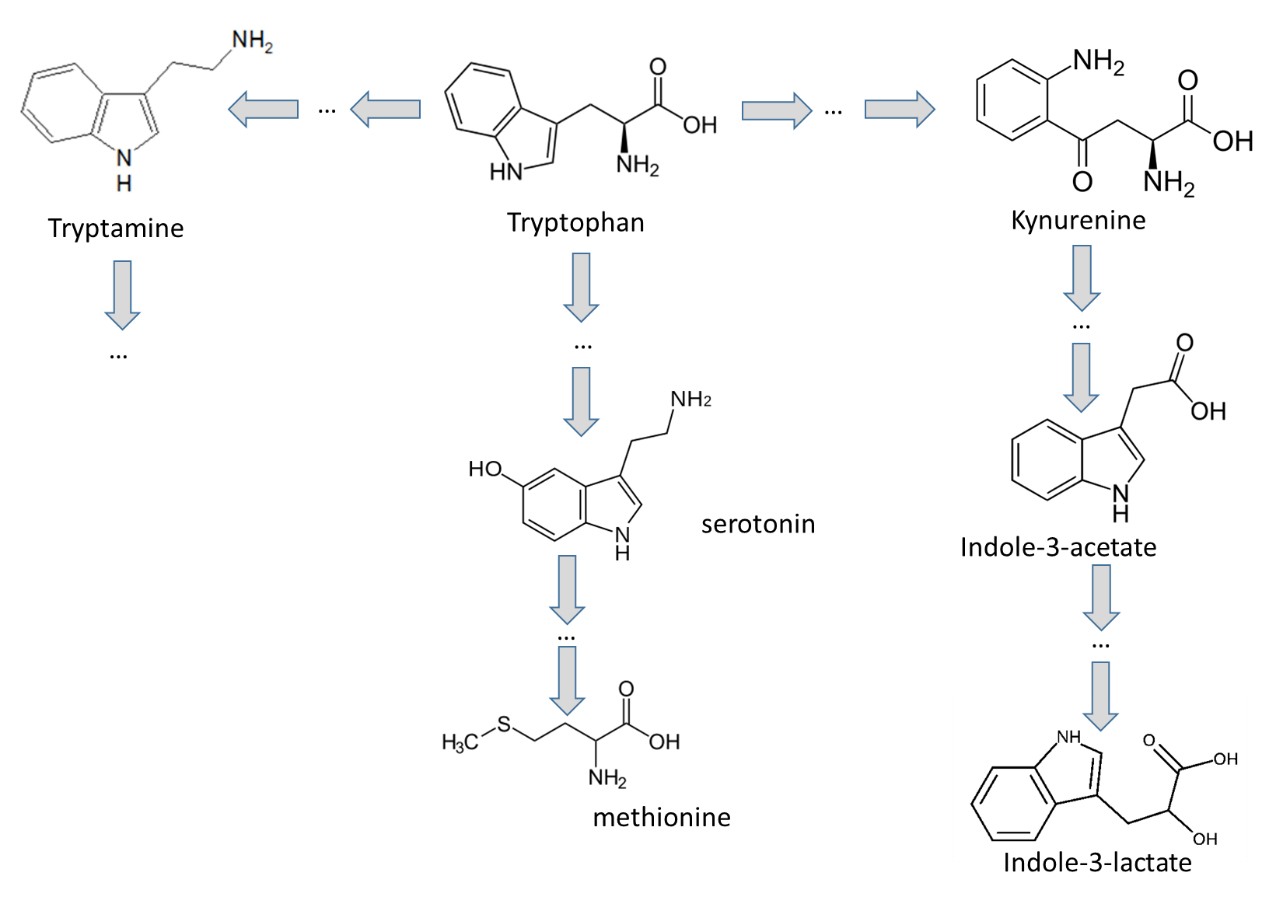
**Supplemental Figure 6.** Abbreviated branches of tryptophan metabolism. ‘…’ represent the presence of intermediate metabolites. Additional detail regarding these pathways can be found in the Tryptophan Metabolism pathway from the Kyoto Encyclopedia of Genes and Genomes (KEGG) pathway (map00380).

Supplementary Table 1. Subject MADRS Scores

| **Subject ID** | **Treatment** | **Study** | **Predose TRT1 MADRS** | **Postdose TRT1 MADRS** | **TRT1 time elapse** | **TRT1 MADRS Change** | **TRT1 MADRS Change (%)** | **Predose TRT2 MADRS** | **Postdose TRT2 MADRS** | **TRT2 time elapse** | **TRT2 MADRS Change** | **TRT2 MADRS Change (%)** |
| --- | --- | --- | --- | --- | --- | --- | --- | --- | --- | --- | --- | --- |
| **13200302** | Esketamine | ESKETIVTRD2001 | 28 | 20 | 2h | -8 | -28.57 | 26 | 24 | 2h | -2 | -7.69 |
| **14900502** | Esketamine | ESKETIVTRD2001 | 31 | 27 | 2h | -4 | -12.9 | 12 | 16 | 2h | 4 | 33.33 |
| **14900303** | Esketamine | ESKETIVTRD2001 | 29 | 6 | 2h | -23 | -79.31 | 29 | 20 | 2h | -9 | -31.03 |
| **14900101** | Esketamine | ESKETIVTRD2001 | 33 | 21 | 2h | -12 | -36.36 | 26 | 25 | 2h | -1 | -3.85 |
| **14900102** | Esketamine | ESKETIVTRD2001 | 23 | 14 | 2h | -9 | -39.13 | 16 | 10 | 2h | -6 | -37.5 |
| **14900503** | Esketamine | ESKETIVTRD2001 | 38 | 17 | 2h | -21 | -55.26 | 15 | 14 | 2h | -1 | -6.67 |
| **14900203** | Esketamine | ESKETIVTRD2001 | 43 | 13 | 2h | -30 | -69.77 | 25 | 7 | 2h | -18 | -72 |
| **13200115** | Esketamine | ESKETIVTRD2001 | 36 | 34 | 2h | -2 | -5.56 | 34 | 32 | 2h | -2 | -5.88 |
| **14900305** | Esketamine | ESKETIVTRD2001 | 34 | 27 | 2h | -7 | -20.59 | 29 | 17 | 2h | -12 | -41.38 |
| **13200112** | Esketamine | ESKETIVTRD2001 | 32 | 17 | 2h | -15 | -46.88 | 1 | 0 | 2h | -1 | -100 |
| **13200113** | Esketamine | ESKETIVTRD2001 | 34 | 34 | 2h | 0 | 0 | 34 | 29 | 2h | -5 | -14.71 |
| **13200108** | Esketamine | ESKETIVTRD2001 | 31 | 2 | 2h | -29 | -93.55 | 19 | 2 | 2h | -17 | -89.47 |
| **13200702** | Esketamine | ESKETIVTRD2001 | 34 | 10 | 2h | -24 | -70.59 | 5 | 2 | 2h | -3 | -60 |
| **13200106** | Esketamine | ESKETIVTRD2001 | 41 | 17 | 2h | -24 | -58.54 | 4 | 4 | 2h | 0 | 0 |
| **13200704** | Esketamine | ESKETIVTRD2001 | 34 | 17 | 2h | -17 | -50 | 8 | 5 | 2h | -3 | -37.5 |
| **13200117** | Esketamine | ESKETIVTRD2001 | 32 | 19 | 2h | -13 | -40.63 | 12 | 0 | 2h | -12 | -100 |
| **13200301** | Esketamine | ESKETIVTRD2001 | 42 | 18 | 2h | -24 | -57.14 | 36 | 18 | 2h | -18 | -50 |
| **14900307** | Esketamine | ESKETIVTRD2001 | 30 | 4 | 2h | -26 | -86.67 | 23 | 16 | 2h | -7 | -30.43 |
| **13200107** | Esketamine | ESKETIVTRD2001 | 32 | 18 | 2h | -14 | -43.75 | 30 | 16 | 2h | -14 | -46.67 |
| **13200105** | Esketamine | ESKETIVTRD2001 | 32 | 13 | 2h | -19 | -59.38 | 2 | 2 | 2h | 0 | 0 |
| **20101310** | Ketamine | KETIVTRD2002 | 37 | 13 | 4d | -24 | -64.86 | 37 | 11 | 14d | -26 | -70.27 |
| **20101210** | Ketamine | KETIVTRD2002 | 33 | 11 | 3d | -22 | -66.67 | 33 | 6 | 14d | -27 | -81.82 |
| **20100811** | Ketamine | KETIVTRD2002 | 34 | 31 | 2d | -3 | -8.82 | 34 | 9 | 14d | -25 | -73.53 |
| **20100813** | Ketamine | KETIVTRD2002 | 34 | 38 | 3d | 4 | 11.76 | 34 | 44 | 13d | 10 | 29.41 |
| **20101318** | Ketamine | KETIVTRD2002 | 33 | 29 | 3d | -4 | -12.12 | 33 | 11 | 15d | -22 | -66.67 |
| **20102404** | Ketamine | KETIVTRD2002 | 32 | 31 | 2d | -1 | -3.13 | 32 | 18 | 14d | -14 | -43.75 |
| **20101215** | Ketamine | KETIVTRD2002 | 35 | 21 | 2d | -14 | -40 | 35 | 3 | 14d | -32 | -91.43 |
| **20100601** | Ketamine | KETIVTRD2002 | 31 | 24 | 3d | -7 | -22.58 | 31 | NA | NA | NA | NA |
| **20100814** | Ketamine | KETIVTRD2002 | 34 | 34 | 1d | 0 | 0 | 34 | 6 | 13d | -28 | -82.35 |
| **20100706** | Ketamine | KETIVTRD2002 | 30 | 25 | 3d | -5 | -16.67 | 30 | 24 | 13d | -6 | -20 |
| **20102201** | Ketamine | KETIVTRD2002 | 36 | 30 | 3d | -6 | -16.67 | 36 | 24 | 14d | -12 | -33.33 |
| **20101316** | Ketamine | KETIVTRD2002 | 37 | 28 | 2d | -9 | -24.32 | 37 | 16 | 14d | -21 | -56.76 |
| **20102405** | Ketamine | KETIVTRD2002 | 30 | 30 | 2d | 0 | 0 | 30 | 12 | 14d | -18 | -60 |
| **20102305** | Ketamine | KETIVTRD2002 | 38 | 15 | 3d | -23 | -60.53 | 38 | 18 | 14d | -20 | -52.63 |
| **20102203** | Ketamine | KETIVTRD2002 | 27 | 25 | 2d | -2 | -7.41 | 27 | 11 | 14d | -16 | -59.26 |
| **20101302** | Ketamine | KETIVTRD2002 | 44 | 25 | 2d | -19 | -43.18 | 44 | 3 | 15d | -41 | -93.18 |
| **20101105** | Ketamine | KETIVTRD2002 | 37 | 36 | 2d | -1 | -2.7 | 37 | 28 | 14d | -9 | -24.32 |
| **20101919** | Ketamine | KETIVTRD2002 | 34 | 23 | 2d | -11 | -32.35 | 34 | 8 | 15d | -26 | -76.47 |
| **20100611** | Ketamine | KETIVTRD2002 | 45 | 34 | 2d | -11 | -24.44 | 45 | 34 | 13d | -11 | -24.44 |
| **20101308** | Ketamine | KETIVTRD2002 | 36 | 31 | 4d | -5 | -13.89 | 36 | 14 | 15d | -22 | -61.11 |
| **20101602** | Ketamine | KETIVTRD2002 | 39 | 21 | 2d | -18 | -46.15 | 39 | 12 | 14d | -27 | -69.23 |
| **20102401** | Ketamine | KETIVTRD2002 | 27 | 19 | 4d | -8 | -29.63 | 27 | 9 | 15d | -18 | -66.67 |
| **20100808** | Ketamine | KETIVTRD2002 | 39 | 18 | 3d | -21 | -53.85 | 39 | 5 | 14d | -34 | -87.18 |
| **20101209** | Ketamine | KETIVTRD2002 | 41 | 30 | 2d | -11 | -26.83 | 41 | 19 | 14d | -22 | -53.66 |
| **20100623** | Ketamine | KETIVTRD2002 | 26 | 12 | 2d | -14 | -53.85 | 26 | 19 | 14d | -7 | -26.92 |
| **20101922** | Ketamine | KETIVTRD2002 | 20 | 14 | 3d | -6 | -30 | 20 | 3 | 14d | -17 | -85 |
| **20102308** | Ketamine | KETIVTRD2002 | 36 | 23 | 1d | -13 | -36.11 | 36 | NA | NA | NA | NA |
| **20101314** | Ketamine | KETIVTRD2002 | 39 | 28 | 2d | -11 | -28.21 | 39 | 27 | 14d | -12 | -30.77 |
| **20101506** | Ketamine | KETIVTRD2002 | 31 | 20 | 4d | -11 | -35.48 | 31 | 20 | 15d | -11 | -35.48 |
| **20100306** | Ketamine | KETIVTRD2002 | 33 | 6 | 2d | -27 | -81.82 | 33 | 8 | 14d | -25 | -75.76 |
| **20100612** | Ketamine | KETIVTRD2002 | 37 | 15 | 2d | -22 | -59.46 | 37 | 1 | 13d | -36 | -97.3 |
| **20101610** | Ketamine | KETIVTRD2002 | 41 | 25 | 10d | -16 | -39.02 | 41 | 24 | 14d | -17 | -41.46 |
| **20102310** | Ketamine | KETIVTRD2002 | 32 | 27 | 3d | -5 | -15.63 | 32 | 28 | 13d | -4 | -12.5 |

**Supplemental Data Files**

SI_File_1.csv: QC'ed GC-TOF metabolite data

SI_File_2.csv: GC-TOF metabolite key

SI_File_3.csv: QC'ed Biocrates Metabolite Data

SI_File_4.csv: Biocrates metabolite key

**References**

1 Singh JB, Fedgchin M, Daly E, Xi L, Melman C, De Bruecker G *et al.* Intravenous esketamine in adult treatment-resistant depression: a double-blind, double-randomization, placebo-controlled study. *Biol Psychiatry*http://www.biologicalpsychiatryjournal.com/article/S0006-3223(15)00914-2/abstract (accessed 9 Nov2015).

2 Scholz M, Fiehn O. SetupX--a public study design database for metabolomic projects. *Pac Symp Biocomput Pac Symp Biocomput* 2007; : 169–180.

3 Fiehn O, Wohlgemuth G, Scholz M, Kind T, Lee DY, Lu Y *et al.* Quality control for plant metabolomics: reporting MSI-compliant studies. *Plant J* 2008; **53**: 691–704.

4 Wikoff WR, Frye RF, Zhu H, Gong Y, Boyle S, Churchill E *et al.* Pharmacometabolomics Reveals Racial Differences in Response to Atenolol Treatment. *PLoS ONE* 2013; **8**: e57639.

5 Ramsay SL, Stoeggl WM, Weinberger KM, Graber A, Guggenbichler W. *Apparatus and method for analyzing a metabolite profile*. EP 1875401 A211-Jan-2007.

6 R Development Core Team. R: A language and environment for statistical computing. R Foundation for Statistical Computing, Vienna, Austria. ISBN 3-900051-07-0, URL http://www.R-project.org/. 2014.http://www.R-project.org.

7 Storey JD, Tibshirani R. Statistical significance for genomewide studies. *Proc Natl Acad Sci* 2003; **100**: 9440–9445.

8 Raivo Kolde. pheatmap: Pretty Heatmaps. R package version 0.7.3.http://CRAN.R-project.org/package=pheatmap. 2012.

9 Oye I, Paulsen O, Maurset A. Effects of ketamine on sensory perception: evidence for a role of N-methyl-D-aspartate receptors. *J Pharmacol Exp Ther* 1992; **260**: 1209–1213.

10 White PF, Ham J, Way WL, Trevor A. Pharmacology of ketamine isomers in surgical patients. *J Am Soc Anesthesiol* 1980; **52**: 231–239.

11 White PF, Schüttler J, Shafer A, Stanski DR, Horai Y, Trevor AJ. Comparative pharmacology of the ketamine isomers Studies in volunteers. *Br J Anaesth* 1985; **57**: 197–203.

12 Torrell H, Montaña E, Abasolo N, Roig B, Gaviria AM, Vilella E *et al.* Mitochondrial DNA (mtDNA) in brain samples from patients with major psychiatric disorders: Gene expression profiles, MtDNA content and presence of the MtDNA common deletion. *Am J Med Genet B Neuropsychiatr Genet* 2013; **162**: 213–223.

13 Shao L, Martin MV, Watson SJ, Schatzberg A, Akil H, Myers RM *et al.* Mitochondrial involvement in psychiatric disorders. *Ann Med* 2008; **40**: 281–295.

14 Jou S-H, Chiu N-Y, Liu C-S. Mitochondrial dysfunction and psychiatric disorders. *Chang Gung Med J* 2009; **32**: 370–379.

15 Chang C-C, Jou S-H, Lin T-T, Lai T-J, Liu C-S. Mitochondria DNA Change and Oxidative Damage in Clinically Stable Patients with Major Depressive Disorder. *PLoS ONE* 2015; **10**: e0125855.

16 Loscalzo J. What We Know and Don’t Know About l-Arginine and NO. *Circulation* 2000; **101**: 2126–2129.

17 Liebenberg N, Joca S, Wegener G. Nitric oxide involvement in the antidepressant-like effect of ketamine in the Flinders sensitive line rat model of depression. *Acta Neuropsychiatr* 2015; **27**: 90–96.

18 Le Mellédo J-M, Mahil N, Baker GB. Nitric oxide: A key player in the relation between cardiovascular disease and major depressive disorder? *J Psychiatry Neurosci* 2004; **29**: 414–416.

19 Gao S-F, Lu Y-R, Shi L-G, Wu X-Y, Sun B, Fu X-Y *et al.* Nitric oxide synthase and nitric oxide alterations in chronically stressed rats: A model for nitric oxide in major depressive disorder. *Psychoneuroendocrinology* 2014; **47**: 136–140.

20 Zarate Jr CA, Machado-Vieira R. Potential Pathways Involved in the Rapid Antidepressant Effects of Nitrous Oxide. *Biol Psychiatry* 2015; **78**: 2–4.

21 Nagele P, Duma A, Kopec M, Gebara MA, Parsoei A, Walker M *et al.* Nitrous Oxide for Treatment-Resistant Major Depression: A Proof-of-Concept Trial. *Biol Psychiatry* 2015; **78**: 10–18.

22 Yu YW-Y, Chen T-J, Wang Y-C, Liou Y-J, Hong C-J, Tsai S-J. Association Analysis for Neuronal Nitric Oxide Synthase Gene Polymorphism with Major Depression and Fluoxetine Response. *Neuropsychobiology* 2003; **47**: 137–140.
